# PHF6 Expression Levels Impact Human Hematopoietic Stem Cell Differentiation

**DOI:** 10.3389/fcell.2020.599472

**Published:** 2020-11-04

**Authors:** Siebe Loontiens, Anne-Catherine Dolens, Steven Strubbe, Inge Van de Walle, Finola E. Moore, Lisa Depestel, Suzanne Vanhauwaert, Filip Matthijssens, David M. Langenau, Frank Speleman, Pieter Van Vlierberghe, Kaat Durinck, Tom Taghon

**Affiliations:** ^1^Cancer Research Institute Ghent (CRIG), Ghent, Belgium; ^2^Department of Biomolecular Medicine, Ghent University, Ghent, Belgium; ^3^Department of Diagnostic Sciences, Ghent University, Ghent, Belgium; ^4^Molecular Pathology and Cancer Center, Massachusetts General Hospital, Boston, MA, United States; ^5^Harvard Stem Cell Institute, Cambridge, MA, United States

**Keywords:** hematopoiesis, PHF6, NOTCH, zebrafish, T cell development

## Abstract

Transcriptional control of hematopoiesis involves complex regulatory networks and functional perturbations in one of these components often results in malignancies. Loss-of-function mutations in *PHF6*, encoding a presumed epigenetic regulator, have been primarily described in T cell acute lymphoblastic leukemia (T-ALL) and the first insights into its function in normal hematopoiesis only recently emerged from mouse modeling experiments. Here, we investigated the role of PHF6 in human blood cell development by performing knockdown studies in cord blood and thymus-derived hematopoietic precursors to evaluate the impact on lineage differentiation in well-established *in vitro* models. Our findings reveal that *PHF6* levels differentially impact the differentiation of human hematopoietic progenitor cells into various blood cell lineages, with prominent effects on lymphoid and erythroid differentiation. We show that loss of PHF6 results in accelerated human T cell development through reduced expression of *NOTCH1* and its downstream target genes. This functional interaction in developing thymocytes was confirmed *in vivo* using a *phf6*-deficient zebrafish model that also displayed accelerated developmental kinetics upon reduced *phf6* or notch1 activation. In summary, our work reveals that appropriate control of *PHF6* expression is important for normal human hematopoiesis and provides clues towards the role of *PHF6* in T-ALL development.

## Introduction

Hematopoietic lineage development is hierarchically organized and involves highly dynamic processes in which hematopoietic stem cells balance between self-renewal and differentiation to generate a wide variety of blood cell types. This process is tightly controlled by various key transcriptional regulators that integrate environmental cues, such as growth factors and cell-intrinsic signals, including epigenetic modifications, to dictate the developmental outcome (Dege and Hagman, 2014; De Obaldia and Bhandoola, 2015; Rothenberg et al., 2016). Many of these developmental genes have been identified through the study of loss- or gain-of-function genetic alterations in hematopoietic malignancies.

The NOTCH signaling pathway is a clear example of a molecular axis that plays a central role in both normal and malignant hematopoiesis. Constitutive NOTCH1 signaling, mainly through *NOTCH1* activating mutations or mutations affecting NOTCH1 pathway regulators, are observed in over 60% of all T cell acute lymphoblastic leukemia (T-ALL) cases (Weng et al., 2004). Further studies subsequently also showed the crucial role of NOTCH1 signaling in normal hematopoiesis with primarily a vital role in normal T cell development (Radtke et al., 1999; Yashiro-Ohtani et al., 2010). NOTCH1 signaling also regulates hematopoietic stem cell (HSC) emergence (Pajcini et al., 2011; Gama-Norton et al., 2015) as well as myeloid (De Obaldia et al., 2013), erythroid (Oh et al., 2013) and lymphoid differentiation (Radtke et al., 2013), highlighting its central regulatory role in hematopoiesis. Over the last decade, multiple factors that work in crosstalk with the NOTCH1 pathway to tightly control normal T cell development have been described and are still a major subject of study, as exemplified by our recent work on the role of GATA3 in the process of T-lineage commitment (Van de Walle et al., 2016).

In addition to *NOTCH1*, *PHF6* is amongst the most frequently affected genes in T-ALL due to loss-of-function mutations (Van Vlierberghe et al., 2010). PHF6, which contains 2 imperfect PHD domains, is considered to be an epigenetic reader molecule (Liu et al., 2015; Todd et al., 2015), exerting its function at least partly through its interaction with components of the NuRD complex such as CHD4 and RBBP7 (Todd and Picketts, 2012). In addition, it affects rRNA synthesis through binding UBF (Wang et al., 2013) and regulates transcription by interacting with the PAF1 transcriptional elongation complex (Zhang et al., 2013). Intriguingly, recent analyses of larger T-ALL cohorts indicate that *PHF6* inactivation predominantly occurs in *NOTCH1* activated T-ALLs, suggesting a functional connection between both genes. This was confirmed by the observation of accelerated leukemia development upon introducing PHF6 mutations in NOTCH1-driven murine T-ALL models, partly by elevating the leukemia stem cell numbers (Hsu et al., 2019; Wendorff et al., 2019).

*PHF6* mutations have not been observed thus far in non-hematopoietic malignancies, suggesting a crucial role in normal hematopoiesis. It is already shown that loss of PHF6 expression in B-ALL cells can induce a partial switch toward the T cell lineage (Soto-Feliciano et al., 2017) and additional recent data support a role for PHF6 in murine hematopoietic stem and progenitor cell homeostasis (McRae et al., 2019) and renewal (Miyagi et al., 2019).

In order to further scrutinize potential roles of PHF6 more broadly during normal human hematopoiesis, we studied the effects of *PHF6* knockdown in normal human hematopoietic precursor cells (HPCs) and validated our observed phenotypes in a *phf6* knock-out zebrafish model (Moore et al., 2012). We show dynamic regulation of PHF6 during normal human hematopoiesis and the requirement of controlled *PHF6* expression to ensure normal hematopoietic lineage differentiation. Furthermore, we show that *PHF6* knockdown during T cell development in human *in vitro* and in zebrafish *in vivo* modulates *NOTCH1* expression and its downstream signaling activity, further supporting a functional interplay between both genes which we believe to be relevant for malignant transformation.

## Materials and Methods

### Isolation of HPCs

Cord blood (CB), peripheral blood (PBL) and pediatric thymus samples were obtained and used according to the guidelines of the Medical Ethical Commission of Ghent University Hospital (Belgium). After lymphoprep density gradient of CB and PBL, mononuclear cells were isolated and used for further purifications. PBL-derived mononuclear cells were labeled with CD3-efluor780 (eBioscience), CD14-FITC (BD Biosciences), CD19-PE (Miltenyi Biotec) and CD56-APC (BD Biosciences) to sort for T cells, monocytes, B cells and NK cells, respectively. CB-derived CD34^+^ cells were purified using magnetic activated cell sorting beads (MACS, Miltenyi Biotec). Subsequently, enriched cord blood CD34^+^ cells were labeled with CD34-PE (Miltenyi Biotec), CD3-APC, CD14-APC, CD19-APC and CD56-APC (APC antibodies from BD Biosciences) to sort CD34^+^Lin^–^ cells with a FACSAriaII (BDIS) (Waegemans et al., 2014). Thymus-derived CD34^+^ T cell precursors were purified using MACS as described (Van de Walle et al., 2013). Purity of the sorted cells was checked on a LSRII (BDIS) and was always >98%.

### Viral Constructs – Transduction of HPCs and Jurkat T-ALL Cells

pLKO.1-puroR (SHC002, control shRNA) and TRCN0000020122 (SHC20122, *PHF6* shRNA) lentiviral vectors were purchased from Sigma in which the puromycin resistance gene was replaced with a PCR-amplified EGFP cDNA using *Bam*HI and *Kpn*I restriction sites. Infectious lentivirus was produced by jetPEI (polyplus transfection^TM^) mediated transfection of the 293FT cell line with either pLKO.1-SHC002-EGFP or pLK0.1-SHC20122-EGFP, in conjunction of the pCMV-VSV-G (envelope) and p8.91 (packaging) constructs. The virus supernatant was harvested 2 and 3 days after transfection. Lentiviral transduction of HPCs was performed on sorted CD34^+^Lin^–^ CB cells or CD34^+^ thymocytes, previously cultured in complete IMDM medium containing 10% FCS and supplemented with TPO (20 ng/ml), SCF (100 ng/ml) and FLT3-L (100 ng/ml) or SCF (10 ng/ml) and IL-7 (10 ng/ml), respectively, for 2 days (cord blood) or one day (thymocytes). 48 h after transduction, cells were harvested and sorted for EGFP^+^ transduced cells. Jurkat T-ALL lymphoblasts were transduced with the same control or *PHF6* shRNA constructs after seeding at a density of 0.5 × 10e^6^ cells/ml in complete RPMI medium containing 10% FCS. Transduced Jurkat cells were harvested 96 h post-transduction for RNA-isolation.

### OP9 Cocultures and Flow Cytometry

Transduced and sorted CD34^+^Lin^–^EGFP^+^ CB cells or CD34^+^EGFP^+^ thymocytes were seeded in a 24 well plate containing a confluent layer of either OP9 control stromal cells expressing GFP (OP9-GFP) or Delta-like-ligand1 (DLL1) or Delta-like-ligand4 (DLL4) expressing OP9 stromal cells (OP9-DLL1 or OP9-DLL4 respectively). All cocultures were performed in α-MEM media (Invitrogen) supplemented with 20% heat-inactivated FCS plus 100 U/ml penicillin, 100 μg/ml streptomycin and 2 mM L-glutamin (all from Invitrogen). To induce and support T and B cell differentiation, cultures were performed in the presence of SCF, IL-7 and FLT3-L (all 5 ng/ml) on OP9-DLL1 and OP9-GFP, respectively. For the generation of NK cells, OP9-GFP cocultures were supplemented with 10 ng/ml IL-15 in addition to SCF, IL-7 and FLT3-L (all 5 ng/ml). For myeloid differentiation, cocultures were executed with SCF, TPO, FLT3-L (all 20 ng/ml) and G-CSF and GM-CSF (both 10 ng/ml). For red blood cell differentiation, cultures were supplemented with SCF, EPO and TPO (50 ng/ml). For y-secretase inhibition (GSI) experiments, 1 μM of 7 N-[*N*-(3,5- difluorophenyl-L-alanyl)]-*S*-phenyl-glycine t-butyl ester (DAPT; Peptides Inter- national, Louisville, KY, United States), diluted in dimethyl sulfoxide (DMSO), was added to the co-cultures and an equal concentration of DMSO was used as control. Cocultures were performed at 37°C in a humidified atmosphere containing 7% (v/v) CO_2_ in air. Cocultures were harvested by forceful pipetting at indicated time points. Obtained cell suspensions were blocked with anti-mouse FcRγII/III (clone 2.4.G2) and human IgG (Fcblock, Miltenyi) to avoid non-specific binding, subsequently stained with combinations of anti-human monoclonal antibodies (BDIS, eBioscience, Biolegend and Miltenyi) and analyzed on a LSRII (BDIS).

### Gene Expression Profiling and Gene Set Enrichment Analysis

RNA samples were profiled on a custom designed Agilent micro-array covering all protein coding genes [33,128 mRNA probes, Human Sureprint G3 8 × 60k micro-arrays (Agilent)] and 12,000 lncRNAs (23,042 unique lncRNA probes) (Volders et al., 2013). The expression datasets generated are deposited in the Gene Expression Omnibus database (GEO) (GSE85373). See Supplementary Methods for further details.

### GSI Treatment in Zebrafish Embryo’s and Imaging

*Tg(rag2:GFP)* embryos were treated from 3 to 6 days post-fertilization (dpf) with 8 μM or 2 μM GSI in E3 media (1X E3 + 0,0001% methylene blue). DMSO was used as control treatment. The embryos were held in a 24 well plate with a maximum of 10 embryos per well and put in fresh E3 media and GSI treatment on a daily basis. Compound treatment was started 3 dpf until day 6 to avoid lethal side-effects during early embryogenesis. For the *phf6* zebrafish studies, *Tg(rag2:GFP)* zebrafish were mated with either wild-type AB zebrafish or *phf6*^c.165del10/c.165del10^ zebrafish. Thymus development and size were monitored and measured from 4 dpf, when rag2 expression commences (Ma et al., 2013), until 6 dpf. The treated embryos were screened for GFP signal by the use of a Nikon SMZ18 microscope. Thymus size was measured with the NIS-Elements Analysis software. During this procedure, the zebrafish were anesthetized with 0.016% tricaine.

## Results

### PHF6 Is Dynamically Expressed During Human Hematopoiesis

**FIGURE 1:**
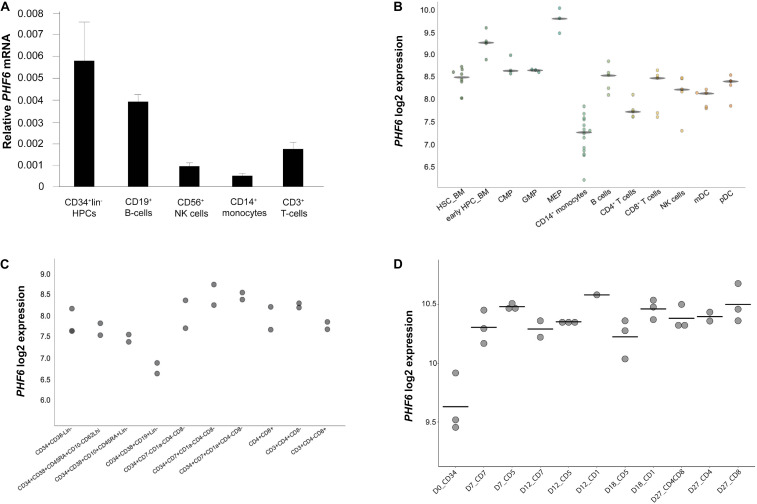
Dynamic *PHF6* expression during human hematopoiesis. Relative expression of *PHF6* in various hematopoietic cell types: **(A)** in sorted subsets of human hematopoietic cell lineages, data shows the average of 3–4 independent samples and error bars indicate SEM; **(B)** adapted from Bloodspot (bloodspot.eu); **(C)** during human lymphopoiesis (Casero et al., 2015) or **(D)** during early T lymphoid development *in vitro* (Canté-Barrett et al., 2017).

To explore the role of *PHF6* in normal human hematopoietic lineage differentiation, we first measured *PHF6* gene expression levels in different human blood cell types (Figure 1A). *PHF6* is expressed in all hematopoietic subpopulations with prominent high expression levels in CD34^+^ hematopoietic precursor cells (HPCs) and CD19^+^ B cells, whereas CD3^+^ T cells showed lower expression. CD56^+^ NK cells and particularly CD14^+^ monocytes displayed the lowest *PHF6* expression levels, in agreement with publicly available data from Bloodspot (bloodspot.eu) (Figure 1B). Furthermore, published expression data during human lymphopoiesis (Figure 1C; Casero et al., 2015) showed a decrease in *PHF6* expression in early B cell precursors compared to in CD34^+^CD38^–^ HPCs but an increase during early T-lymphoid development. The latter was also observed in *in vitro* generated T cell precursors on OP9-DLL1 stromal layers, indicating that this *in vitro* model recapitulates *PHF6* expression dynamics that occur *in vivo* (Figure 1D; Canté-Barrett et al., 2017). Thus, *PHF6* is dynamically expressed during human hematopoiesis which suggests regulatory functions during hematopoietic stem cell differentiation.

### Reduced PHF6 Expression Impacts on Erythroid and Lymphoid Differentiation

**FIGURE 2:**
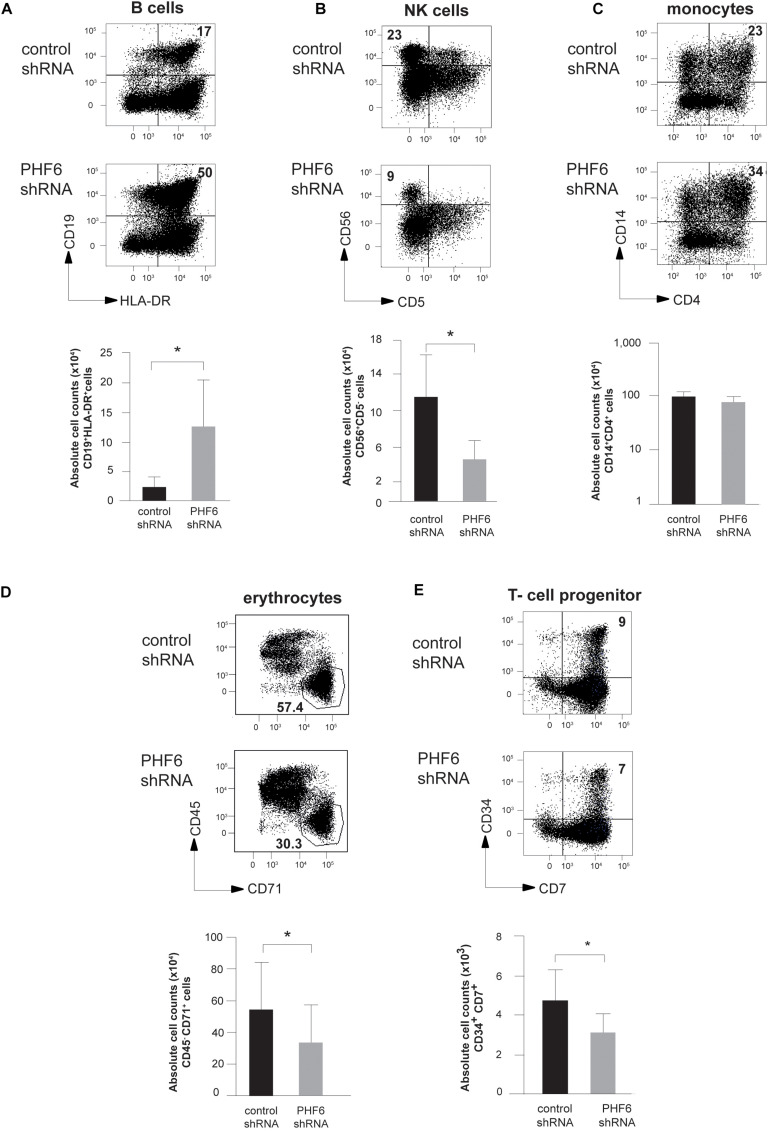
PHF6 is essential for normal hematopoietic differentiation. **(A–D)** (*up*) Dot plots show flow cytometry analysis of control and *PHF6* shRNA transduced cord blood CD34^+^Lin^–^ precursors in OP9-GFP cocultures, showing the development of **(A)** CD19^+^HLA-DR^+^ B-lineage cells after 28 days of coculture, **(B)** CD56 + CD5- NK cells after 21 days of coculture, **(C)** CD14^+^ CD4^+^ monocytes after 14 days of coculture and **(D)** CD45^+^ CD71^–^ erythrocytes after 7 days of coculture. Bar plots (*down*) show absolute numbers of corresponding populations. **(E)** (*up*) Dot plots show flow cytometry analysis of control and *PHF6* shRNA transduced cord blood CD34^+^Lin^–^ precursors in OP9-DLL1 cocultures, showing the development of CD34^+^CD7^+^ T cell precursors after 7 days of coculture. Bar plot (*down*) shows absolute numbers of the corresponding population. Data shows average of 5–7 independent experiments and error bars indicate SEM. *P < 0.05 (non-parametric paired Wilcoxon test).

To assess the impact of *PHF6* loss on the differentiation potential of human HPCs, we first optimized stable *PHF6* knockdown in Jurkat T-ALL cells using lentiviral gene transfer (Supplementary Figure 1a) and this confirmed strong PHF6 downregulation both at the mRNA (*left*) and protein level (*right*) which was also observed at the mRNA level in cord blood (CB)-derived CD34^+^ HPCs (Supplementary Figure 1b). Next, *PHF6* shRNA transduced CD34^+^Lin^–^ CB HPCs were induced to differentiate *in vitro* in the presence of lineage-specific cytokines using well-established OP9 cocultures (Van de Walle et al., 2011). This revealed marked effects of reduced PHF6 levels on human hematopoiesis. In OP9-GFP cultures conditions that permit B cell development, we noticed a significant increase in both the frequency and absolute number (Figure 2A) of CD19^+^HLA-DR^+^ B cells upon *PHF6* knockdown compared to the control condition. In contrast, reduced *PHF6* expression in CD34^+^ CB HPCs that were cultured in NK-lineage conditions significantly decreased the generation of CD56^+^CD5^–^ NK cells compared to control transduced cells, both in frequency and absolute numbers (Figure 2B). In myeloid culture conditions (Klinakis et al., 2011; Van de Walle et al., 2011), the impact of *PHF6* knockdown on monocyte differentiation was modest. A slight but consistent increase in the frequency of CD14^+^CD4^+^ monocytes was observed, but no significant difference was seen in absolute cell counts (Figure 2C). Given the high *PHF6* expression in MEPs (Figure 1B), we also evaluated the effect of *PHF6* downregulation on the differentiation of CD34^+^ CB HPCs toward erythrocytes and observed a significant decrease in the generation of CD71^+^CD45^–^ red blood cells compared to control transduced precursors (Figure 2D). On OP9-DLL1 stromal cells that permit induction of T-lineage differentiation, a small but consistent reduction in the most immature CD34^+^CD7^+^ T cell precursors was observed upon *PHF6* knockdown which resulted in a significant reduction in their absolute numbers compared to the control (Figure 2E).

**FIGURE 3:**
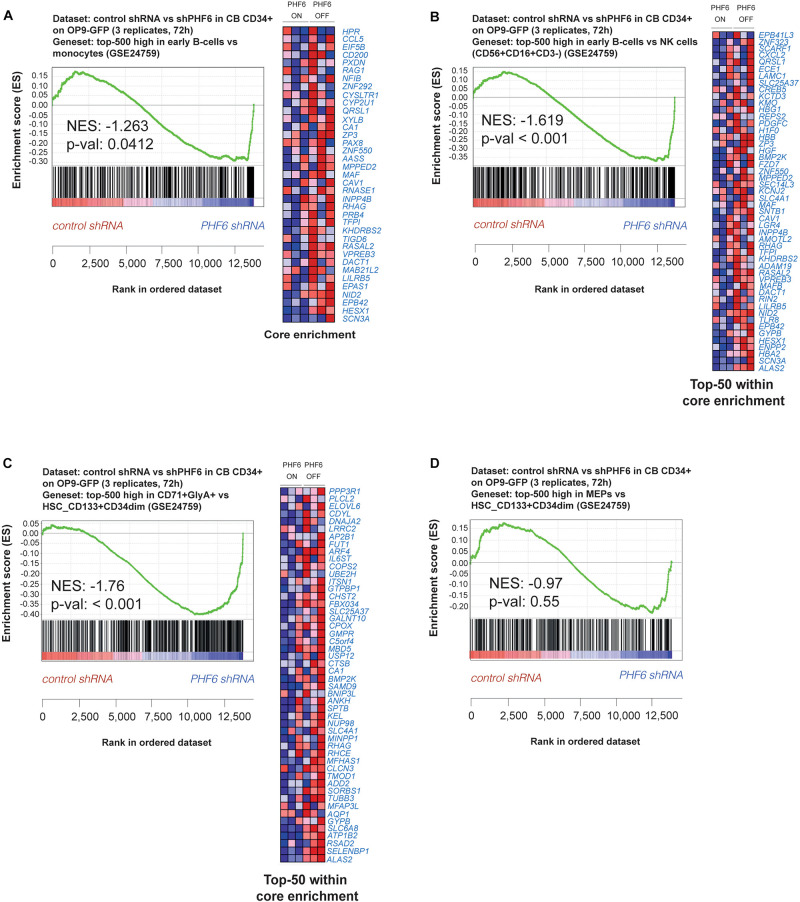
PHF6 controls the expression of hematopoietic lineage genes. **(A,B)** Gene Set Enrichment Analysis shows that an early B cell gene signature is significantly enriched at expense of **(A)** a myeloid gene signature (GSE24759) and **(B)** NK cell (CD56^+^CD16^+^CD3^–^) gene signature (GSE24759) in short-term cultures of CD34^+^ progenitors on an OP9-GFP stromal feeder layer with stable PHF6 knockdown (GSE85373). **(C,D)** Gene Set Enrichment Analysis shows that an erythrocyte gene signature **(C)** but not a MEP gene signature **(D)** is significantly enriched at expense of a HSCs gene signature (GSE24759) in short-term cultures of CD34^+^ progenitors on an OP9-GFP stromal feeder layer with stable PHF6 knockdown.

To understand the potential underlying mechanisms of these developmental changes, we performed gene expression profiling in shPHF6 transduced CD34^+^ progenitor cells that were short-term cultured on OP9-GFP and applied gene set enrichment analysis (GSEA) based on the resulting expression signatures and publically available transcriptional profiles of sorted populations of different human hematopoietic cell types (Van de Walle et al., 2013). Consistent with the preferential differentiation toward B lymphocytes of *PHF6*-deficient HPCs, B cell lineage genes (including *VPREB3*, *RAG1* and *CD200*) were significantly enriched in the set of genes upregulated upon *PHF6* knockdown compared to myeloid (Figure 3A) or NK cell signatures (Figure 3B). However, despite the reduction in red blood cell development, an enrichment in genes expressed higher in erythrocytes compared to HSCs was observed upon *PHF6* knockdown (Figure 3C), but not for genes that are higher expressed in MEPs compared to HSCs (Figure 3D). Taken together, our data indicates that *PHF6* levels modulate human lymphoid development and suggests that loss of *PHF6* expression results in premature induction of erythroid genes that hampers their development.

### PHF6 Modulates *NOTCH1* Expression and Its Downstream Signaling Activity in Human T-Lineage Cells

**FIGURE 4:**
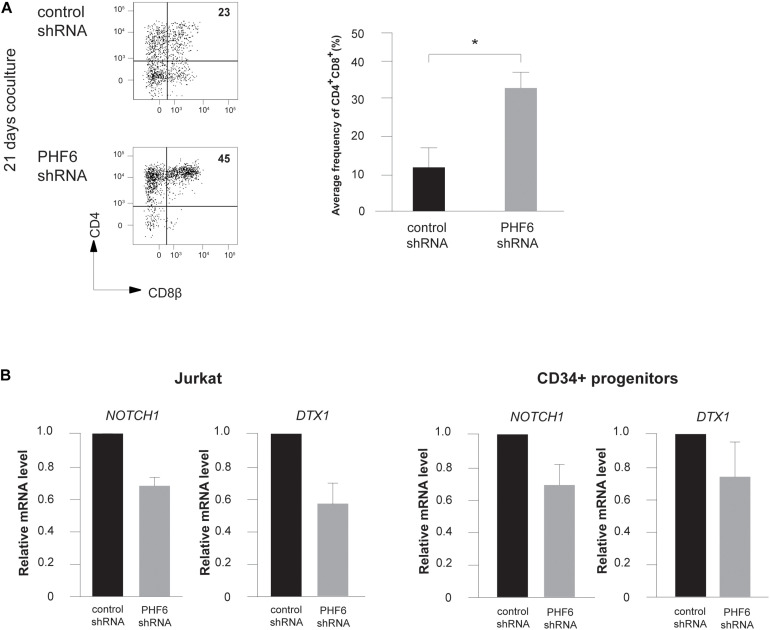
PHF6 modulates Notch1 expression and its downstream signaling activity. **(A)** (*left*) Flow cytometry analysis of control and *PHF6* shRNA transduced CD34^+^ thymocytes in OP9-DLL1 cocultures in the presence of IL7, SCF and FLT3L, showing the development of CD4^+^CD8β^+^ DP thymocytes after 21 days of coculture. (*right*) Bar plot showing the frequency of CD4^+^CD8β^+^ DP thymocytes, generated in the corresponding cultures. Data shows the average of 4 independent experiments and errors bars show SEM. *P < 0.05 (paired *t*-test) **(B)** Normalized *NOTCH1* and *DTX1* expression in Jurkat cells (left) and CB-derived CD34^+^ HPCs (right) following control or *PHF6* shRNA transduction as indicated. Data shows the average expression in 3 independent samples and error bars indicate SEM.

**FIGURE 5:**
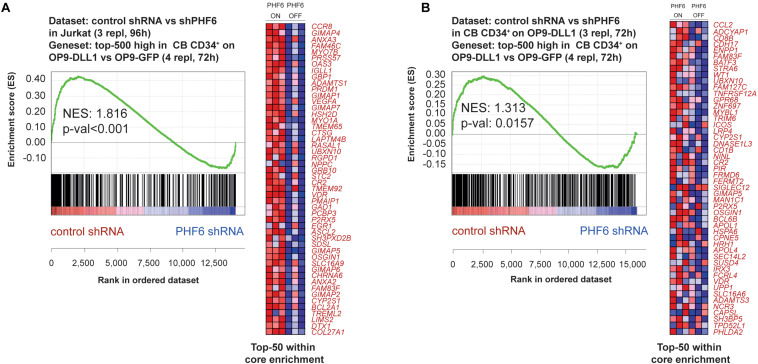
PHF6 modulates the Notch1 gene signature. Gene Set Enrichment Analysis shows that the top-500 significantly induced genes in CB CD34^+^ progenitors cultured on an OP9-DLL1 stromal feeder layer in comparison to OP9-GFP cocultures are significantly enriched in the set of genes that are downregulated upon stable knockdown of PHF6 in panel **(A)** Jurkat T-ALL cells (GSE85373) and **(B)** CB CD34^+^ cells cultured on an OP9-DLL1 stromal feeder layer (GSE85373).

Given the central role of NOTCH1 during early T cell development and the frequent co-occurrence of activating *NOTCH1* and loss-of-function *PHF6* mutations in T-ALL, we evaluated the effects of *PHF6* knockdown on T cell development in more detail. Indeed, the increase in B-lineage differentiation (Figure 2A) and reduction in early T-lymphoid development (Figure 2E) are suggestive for reduced Notch1 activity upon *PHF6* knockdown. Therefore, we initiated OP9-DLL1 cocultures with control or *PHF6* shRNA transduced human CD34^+^ thymocytes and observed remarkable accelerated differentiation toward the CD4^+^CD8^+^ double positive (DP) differentiation stage (Figure 4A). Interestingly, such enhanced DP differentiation was previously also observed in CD34^+^ human thymocytes upon attenuation of NOTCH activity (Van de Walle et al., 2013, 2009), indicating that *PHF6* knockdown may indeed negatively impact NOTCH1 signaling. Indeed, consistent with the PHF6-mediated regulation of *NOTCH1* expression in B-ALL cells (Meacham et al., 2015), we also observed reduced *NOTCH1* expression upon stable PHF6 knockdown in Jurkat leukemic T cells, as well as in OP9-DLL1 cocultured human CD34^+^ HPCs (Figure 4B). This also affected downstream NOTCH1 signaling as shown by the concomitant reduced expression of the direct NOTCH1 target gene *DTX1* (Figure 4B). In addition we found a significant enrichment of genes reported as NOTCH1-dependent in CD34^+^ T cell progenitors (Durinck et al., 2014) in the set of downregulated genes upon shPHF6 knockdown in Jurkat cells (Figure 5A) and in CD34^+^ T cell precursors (Figure 5B). Importantly, these included canonical target genes that are NOTCH1-dependent during early stages of human T cell development such as *DTX1* and *HES4* (Supplementary Table 1). These observations were confirmed independently in ALL-SILL T-ALL lymphoblasts. To this end, we transiently downregulated *PHF6* by means of siRNA-mediated transfection (Supplementary Figure 2a), resulting in a downregulation of *NOTCH1* and *DTX1* expression (Supplementary Figures 2b,c). As shown by GSEA, also *NOTCH1* downstream target genes are significantly downregulated upon *PHF6* knockdown (Supplementary Figure 2d). Overall, these finding establish that loss of *PHF6* not only reduces *NOTCH1* expression itself, but that it also functionally affects NOTCH1 downstream signaling.

### PHF6 Levels Modulate NOTCH Activity During Human T Cell Development

**FIGURE 6:**
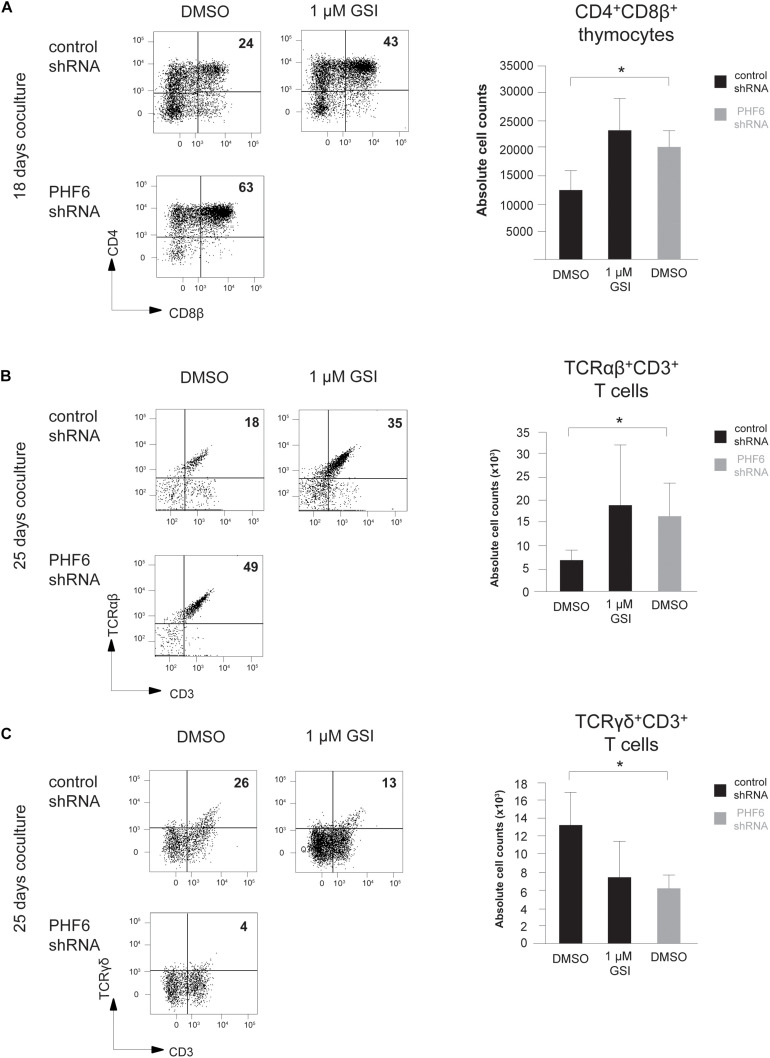
Loss of PHF6 mimics reduced Notch activity during human T cell development. **(A–C)** (*left*) Flow cytometry analysis and (*right*) absolute cell counts of control versus *PHF6* shRNA transduced or DMSO versus 1 μM GSI treated CD34^+^ thymocytes in OP9-DLL1 cocultures showing **(A)** the development of CD4^+^CD8β^+^ DP thymocytes after 18 days of coculture, **(B)** the development of CD3^+^TCRαβ^+^ thymocytes after 25 days of coculture and **(C)** the development of CD3^+^TCRγδ^+^ thymocytes after 25 days of coculture. Data shows the average of 3 independent experiments and errors bars show SEM. *P < 0.05 (paired *t*-test).

To further unravel the connection between PHF6 and NOTCH1 during thymopoiesis, we compared the effects of modulated *PHF6* expression and altered NOTCH1 signaling activity in more detail by stable knockdown of *PHF6* and pharmacological inhibition of Notch signaling using a γ-secretase inhibitor (GSI), respectively. Here, and to confirm our OP9-DLL1 derived results, we used OP9-DLL4 cocultures to provide the physiological NOTCH1 ligand. Similar as on OP9-DLL1 cocultures, thymocytes with reduced PHF6 levels progressed significantly faster toward the double positive (CD4^+^CD8^+^, DP) stage of T cell development in comparison to control transduced cells after 18 days of culture (Figure 6A). These increased numbers of DP cells are most likely to arise due to a general accelerated differentiation, given that this rise in DP frequency and absolute counts upon PHF6 knockdown compared to controls is consistently, also already observed at earlier time points of 6 and 14 days of coculture (Supplementary Figures 3a,b). Furthermore, a comparable acceleration towards this DP stage was also observed in control transduced T cell precursors exposed to GSI both in frequency and absolute cell counts (Figure 6A and Supplementary Figures 3a,b), in agreement with previous findings (Van de Walle et al., 2009). Consistent with this DP phenotype and the differential requirement for NOTCH signaling activity (Van de Walle et al., 2009, 2013), loss of PHF6 skewed differentiation of human T cell precursors toward TCR-αβ T cells (Figure 6B) at the expense of TCR-γδ T cell development (Figure 6C) both in frequencies as in absolute counts respectively, further supporting a functional parallel between NOTCH1 and PHF6 during normal T cell development.

### The Phf6-Notch Regulatory Axis During Early T Cell Development Is Conserved Between Zebrafish and Human

Given the important differences in the role of NOTCH signaling during early mouse and human T cell development (Taghon et al., 2012), we evaluated whether zebrafish could be a more appropriate model to confirm our *in vitro* human PHF6 data in an *in vivo* context. Moreover, the transparent embryos of this model organism additionally allow to visualize developmental processes.

Sequence alignment of the Phf6 protein for human, zebrafish, mouse, chicken, chimpanzee and rat revealed a high degree of amino acid sequence conservation across these different species (Supplementary Figure 4a). Blasting (NCBI protein blast) the human PHF6 protein sequence against that of zebrafish resulted in an overall 71% peptide identity with an even higher 81.1 and 92.5% identity for both functional plant homeodomain zinc fingers 1 and 2 (PHD1 & PHD2), thus indicating a high degree of functional conservation. In human, the highest *PHF6* expression is observed in the thymus, ovary and thyroid tissues while a moderate expression is detected in spleen, testis and adipose tissue (Van Vlierberghe et al., 2010). In zebrafish, we observed *phf6* expression in all dissected organs with the highest expression in ovary, testis, kidney and thymus, thus closely resembling the human expression pattern (Supplementary Figure 4b). The expression of *phf6* in the zebrafish kidney is particularly interesting since all hematopoietic cells types, except for thymocytes, are found in this organ (Jing and Zon, 2011; Jagannathan-Bogdan and Zon, 2013). Thus, in addition to high structural conservation, these findings also suggest functional conservation of PHF6 in zebrafish and human.

**FIGURE 7:**
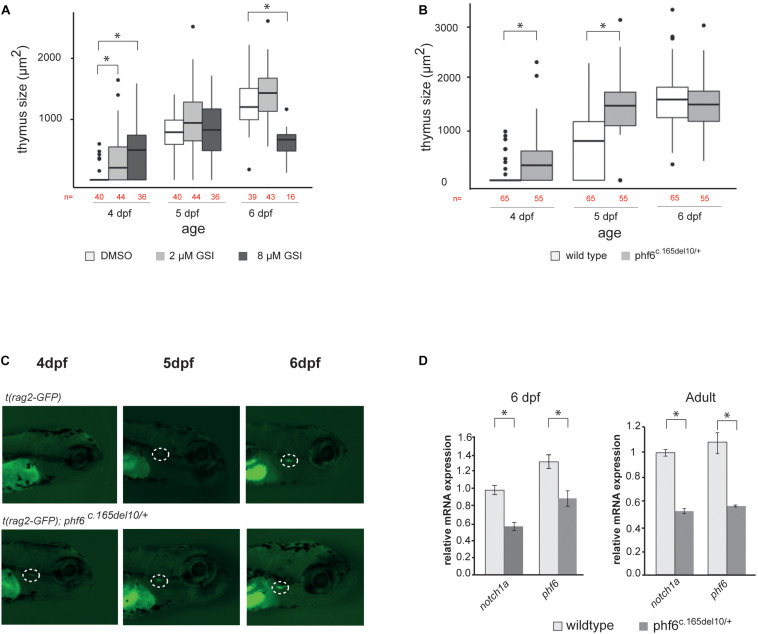
*phf6* downregulation accelerates T cell development *in vivo*. **(A)** Box plot showing thymus size (μm^2^) at 4, 5, and 6 days post-fertilization (dpf) based on GFP signal of wild type (AB) fish treated with 2 or 8 μM of gamma-secretase inhibitor (GSI) or DSMO as control treatment. Details on these results are provided in Supplementary Table 2 and statistical analysis is shown in Supplementary Table 3, ^∗^*P* < 0.05 (Wilcoxon rank sum test). **(B)** Boxplot showing thymus size (μm^2^) of wild type (AB) and phf6^c.165del10/+^ heterozygous embryos from 4 until 6 dpf based on rag2-GFP signal quantification. ^∗^*P* < 0.05 (Wilcoxon rank sum test, Supplementary Table 4). **(C)** Representative image of thymus visualization used for quantification of data as shown in panel **(A).** Original magnification X30. Circle with white dashed line indicates emerging thymus. **(D)** Average normalized *notch1a* and *phf6* expression in sorted T cells of 4 replicates of 100 pooled wild type (AB) and *phf*6^c.165*del10*⁣/ +^ embryo’s on 6dp (left) and of 3 replicates of 6 pooled wildtype and 6 pooled *phf6*^c.165*del10*⁣/ +^ adult zebrafish (right). Error bars indicate SEM, ^∗^*P* < 0.05 (unpaired *T*-test).

To validate such functional similarities between human and zebrafish regarding NOTCH signaling, *Tg(rag2-GFP)* fish were treated with either low (2 μM, *n* = 44) or high doses (8 μM, *n* = 36) of GSI compared to the DMSO (*n* = 40) solvent control. *Tg*(*rag2*-*GFP*) zebrafish were used because this approach allows visualization of the emerging thymus through the *rag2*-expressing GFP^+^ thymocytes. T cell development was monitored, and thymus size measured from 4 days post fertilization (dpf) until 6 dpf. Consistent with our findings *in vitro*, zebrafish treated with 2 μM GSI displayed accelerated T cell maturation compared to the control treated fish. At 4 dpf 59% (2 μM) and 72% (8 μM) GSI-treated fish showed GFP signal in the thymus while only 15% of the DMSO control treated fish had visible GFP expression in the thymus (Figure 7A and Supplementary Tables 2, 3). Applying a higher dose of GSI (8 μM) resulted initially in faster T cell developmental kinetics at 4 dpf, but significantly reduced the thymus size in later stages of development at 6 dpf, consistent with our human data (Taghon et al., 2012) (Figure 6A and Supplementary Tables 2, 3). Collectively, the zebrafish data support conservation of Phf6 function and of Notch signaling during early T cell development between zebrafish and human.

### Phf6 Controls *Notch1* Expression and T Cell Development *in vivo*

To assess a role for Phf6 in T cell development *in vivo*, we took advantage of an available *phf6* TALEN mutated zebrafish line (Moore et al., 2012; Loontiens et al., 2020). Specifically, *phf6* mutations were created in exon 2 and resulted in a 10 nucleotide deletion and premature stop codon (Supplementary Figure 4c). The effect of the 10 bp deletion in the *phf6* gene was analyzed by means of RT-qPCR, and a significant downregulation of *phf6* levels was seen in *phf6* heterozygote fish compared to wildtype, mimicking PHF6 downregulation seen upon knockdown *in vitro* (Supplementary Figure 4d). We then further investigated the role of Phf6 in thymopoiesis *in vivo* by crossing transgenic *Tg(rag2:GFP)* zebrafish with either AB wildtype or *phf6*^c.165del10/c.165del10^ mutant zebrafish. The resulting offspring were kept separately at 4 dpf and thymus development was monitored until 6 dpf (Figures 7B,C). At 4 dpf, significantly more *phf6*^c.165del10/+^ mutant fish showed GFP expression in the thymus region compared to the AB wild type (62 versus 14%, respectively, Figures 7B,C and Supplementary Table 4). By 5 dpf, almost all (96%) of the *phf6*^c.165del10/+^ mutant fish displayed detectable *rag2-*GFP expression while the wild type fish needed 6 days to reach such frequencies (Figures 7B,C and Supplementary Table 4). Independent morpholino experiments confirmed these Phf6-mediated effects on T cell development (Supplementary Figure 4e). Quantification of thymus size revealed a significant larger thymus at 4 and 5 dpf for *phf6*^c.165del10/+^ mutant compared to wildtype fish (Figure 7B and Supplementary Table 4), indicating that *phf6* downregulation results in initial faster kinetics of T cell development. In agreement, RT-qPCR analysis of sorted thymocytes (Loontiens et al., 2019) showed a downregulation of *notch1a* expression in *phf6*^c.165del10/+^ embryos compared to thymocytes from wild type embryo’s (Figure 7D), as well as in thymocytes of adult *phf6*^c.165del10/+^ versus wild type zebrafish (Figure 7D). Collectively, these *in vivo* findings confirm that Phf6 modulates Notch1 expression during thymopoiesis and that loss of Phf6 mimics the effects of reduced Notch1 expression on thymopoiesis in zebrafish, similarly as observed in human.

## Discussion

Despite the fact that loss-of-function *PHF6* mutations are amongst the most frequent genetic alterations in T-ALL, the role of PHF6 in normal and malignant hematopoiesis is functionally still uncharacterized. Here, we show that *PHF6* is dynamically expressed during hematopoiesis and that *PHF6* levels functionally impact normal human hematopoietic lineage differentiation. Consistent with the strong association between loss-of-function *PHF6* mutations and activating *NOTCH1* mutations in T-ALL (Wang et al., 2011; Li et al., 2016), we reveal that *NOTCH1* expression and signaling is dependent on *PHF6* expression during normal T cell development. We observed similar developmental effects for both PHF6 and Notch activity in our *in vitro PHF6* loss-of-function experiments for human T cell development. In addition, we independently confirmed these findings *in vivo* using a *pfh6* knock-out zebrafish model.

Importantly, the findings from this manuscript are consistent with our previous work on the role of Notch signaling during human T cell development. The differentiation of human CD34^+^ thymocytes into DP and TCR αβ-lineage thymocytes is indeed dependent on a reduction of Notch signaling activity in the OP9-DLL1 (or OP9-DLL4) coculture system that can be triggered by adding low dosages of GSI (Taghon et al., 2012). *In vivo*, this reduction in Notch activation is equally important (Durinck et al., 2014), as also illustrated by the induction of T-ALL upon continuous Notch activation (Ferrando, 2009), and this could be controlled through migration within the thymic microenvironment to regions with a reduced availability of Notch ligands (Van de Walle et al., 2013). Also the reduction in TCRγδ T cell development that occurs upon knockdown of PHF6 corresponds with the strong Notch requirement for their development (Van de Walle et al., 2009, 2013). We recently illustrated that Notch activity during early human T cell development is also dependent on GATA3 activity that mediates downregulation of *DTX1* (Van de Walle et al., 2016), consistent with the impact of reduced *PHF6* expression on *NOTCH1* and *DTX1* expression. Intriguingly, besides a direct connection between PHF6 and NOTCH1 as shown by the binding of PHF6 to the transcription start site locus of NOTCH1 in Jurkat cells (Meacham et al., 2015), recent data have shown that loss of PHF6 function also increases chromatin accessibility at the GATA3 locus (Soto-Feliciano et al., 2017), suggesting that the observed effects of *PHF6* knockdown on T cell development may be mediated by several transcriptional mediators in addition to changes in NOTCH1.

While it remains to be determined if PHF6 has a similar role in mice, we choose not to use this organism for *in vivo* experiments given the significant differences between mouse and human regarding the Notch signaling requirements during T cell development (Taghon et al., 2012). Indeed, while Notch signaling is important in both mouse and human to induce T-cell lineage specification in multipotent hematopoietic precursors (Wilson et al., 2001; Van de Walle et al., 2009), the subsequent stages of T cell development show altered Notch signaling requirements in both species. While differentiation of specified T cell precursors into DP TCR-αβ lineage cells is highly Notch-dependent in mice (Ciofani et al., 2006; Taghon et al., 2006), this developmental process occurs much more efficient in human when Notch signaling is reduced (Van de Walle et al., 2009, 2013; Dolens et al., 2020). In contrast, TCRγδ T cell development is highly Notch-dependent in human (Van de Walle et al., 2009, 2013; Dolens et al., 2020) but less in mouse (Ciofani et al., 2006; Taghon et al., 2006). In addition, genetic studies in zebrafish are increasingly used to study human genetic aberrations, as illustrated for *BCL11B* mutations during T cell development (Punwani et al., 2016). The findings in this manuscript further support the importance of zebrafish as a model to study normal hematopoiesis and particularly T cell development (Zhang and Wiest, 2016) as both the effects of *PHF6* knockdown and of reduced NOTCH signaling activation on human T cell development were confirmed *in vivo*. The possibility to image T cell development in embryos also allows to perform kinetic studies *in vivo*, which is also a significant strength of *in vitro* studies, since they allow to reveal developmental abnormalities that may be obscured in adult organisms as a result of homeostatic expansion mechanisms. However, in zebrafish, thymopoiesis consists out of two waves, with the first wave starting from 3 dpf and mainly producing αβ-CD4 T cells, while the second wave commences at 8 dpf and gives rise to αβ-CD4, αβ-CD8 and γδ T cells (Tian et al., 2017). This study suggests that both Phf6 and Notch influence this first wave of thymopoiesis, producing the αβ-CD4 T cells. The enhanced TCR-αβ differentiation at the expense of the γδ-lineage could not be assessed by our experimental timeframe and set up. Additional studies are required to fully explore to what extent hematopoiesis is conserved between human and zebrafish as compared to mice (Mestas and Hughes, 2004; Payne and Crooks, 2007).

The observed effect of *PHF6* knockdown on B cell development is also in line with reduced NOTCH1 signaling activity since B-lineage differentiation is extremely sensitive to small dosages of signaling activity. Although these experiments were performed on OP9-GFP stromal cells in the absence of a NOTCH ligand that is sufficiently strong to induce T cell development and to fully inhibit B cell development, OP9 cells express significant levels of Jagged-1 and -2 (Schmitt and Zúñiga-Pflücker, 2002) that can partially hamper B cell development (Van de Walle et al., 2011). However, given the significant increase in B cell signature genes upon *PHF6* knockdown, it is possible that PHF6 directly regulates at least some of these genes independent of Notch signaling activity, consistent with its role as an epigenetic regulator (Soto-Feliciano et al., 2017). Such a direct regulatory role for PHF6 may also be required for the expression of NK-lineage genes that are not Notch-dependent in all circumstances, such as *DTX1* and *TBX21* (Rankin et al., 2013), although we have also demonstrated that Notch signaling enhances NK cell development (De Smedt et al., 2007). While we mainly focused on lymphocyte development, the lack of effect on monocytic differentiation upon PHF6 knockdown and the low expression that is observed in *ex vivo* isolated monocytes suggests that PHF6 is mainly involved in regulating lymphocyte development. Nevertheless, we also observed a clear phenotype with respect to erythroid development. Although the impact of reduced PHF6 expression was apparently inconsistent with respect to the reduced development of erythrocytes compared to the increased expression of red blood cell genes, the premature expression of these genes in HPCs may hamper the appropriate differentiation of these precursors toward erythrocytes given that genes from the intermediate MEP stage were not altered upon *PHF6* knockdown. Intriguingly, also Notch activation has been implicated in erythroid development in mice (Oh et al., 2013).

In the context of leukemia, PHF6 has been suggested to act as an oncogene in B-ALL (Meacham et al., 2015; Soto-Feliciano et al., 2017). Although the enhanced differentiation of CD34^+^ HPCs towards the B cell lineage upon *PHF6* knockdown might seem inconsistent with such an oncogenic role, it is well-established that context-specific roles may alter protein function and/or requirement. This has for instance been clearly illustrated for Notch activation that strongly antagonizes early B cell development (Wilson et al., 2001), yet, Notch2 activation is later essential within the B cell lineage for marginal zone B cell development (Saito et al., 2003). Moreover, activating Notch mutations have been described in B cell malignancies (Chiang et al., 2016). In T-ALL, *PHF6* and *NOTCH1* are considered to act as tumor suppressor and oncogenes, respectively (Ferrando, 2009; Van Vlierberghe et al., 2010). Therefore, our finding that *PHF6* knockdown attenuates NOTCH1 activity may seem counterintuitive in the context of T-ALL oncogenesis. However, in T-ALL patients that harbor loss-of-function *PHF6* mutations, there is a significant association with activating mutations in *NOTCH1* or its downstream target *IL7R* (Wang et al., 2011; Vicente et al., 2015; Li et al., 2016), indicating that reduced PHF6 function triggers the cells to compensate for the reduced activity of these critical T-lineage proliferation/survival pathways.

In conclusion, our results reveal an important regulatory role for PHF6 during normal hematopoiesis and provide novel clues towards the tumor suppressor role of *PHF6* in T-ALL.

## Data Availability Statement

The datasets presented in this study can be found in online repositories. The names of the repository/repositories and accession number(s) can be found below: https://www.ncbi.nlm.nih.gov/geo/, GSE85373.

## Ethics Statement

The studies involving human participants were reviewed and approved by the Medical Ethical Commission of Ghent University Hospital (Belgium). Written informed consent to participate in this study was provided by the participants’ legal guardian/next of kin. The animal study was reviewed and approved by Massachusetts General Hospital Subcommittee on Research Animal Care (OLAW Assurance # A3596-01 under protocol #2011N000127) and by the Ghent University Committee on Ethics of Animal Experiments (Ghent University Hospital, Ghent, Belgium; Permit Number: ECD 11/37).

## Author Contributions

KD, SL, AC-D, SS, and IV performed the experiments, analyzed the data, and wrote the manuscript. FEM performed the experiments and analyzed the data. SV, LD, and FM performed the experiments. DL provided reagents and intellectual guidance. PV, FS, and TT designed the research, analyzed the data, and wrote the manuscript. All authors contributed to the article and approved the submitted version.

## Conflict of Interest

The authors declare that the research was conducted in the absence of any commercial or financial relationships that could be construed as a potential conflict of interest. The handling editor declared a shared affiliation with several of the authors, FM and DL, at the time of review.
