# Supplementary material for: PHF6 Expression Levels Impact Human Hematopoietic Stem Cell Differentiation
Source: Front Cell Dev Biol. 2020 Nov 4;8:599472. doi: 10.3389/fcell.2020.599472 (PMC7672048; doi:10.3389/fcell.2020.599472)
Supplement: Supplementary file 1 [file Data_Sheet_1.pdf]

Supplementary Figure Legends

Supplementary Figure 1: (**a**) Normalized PHF6 mRNA and protein expression in Jurkat T-ALL cells upon stable transduction with either a control or *PHF6* targeting shRNA, resulting in PHF6 downregulation >80%. (**b**) Normalized *PHF6* expression in human cord blood $CD34^+$ cells, transduced with either control or *PHF6* targeting shRNA. Data show the average expression in 7 independent samples and error bars indicate SEM.

Supplementary Figure 2: (**a**) Normalized PHF6 protein expression in ALL-SIL T-ALL cells upon siRNA based PHF6 knock down (96 h) by 2 different siRNA's, resulting in PHF6 downregulation >75% compared to a non-targeting control. (**b**) RT-qPCR analysis showing normalized *NOTCH1* and *DTX1* expression in ALL-SIL cells following control or *PHF6* siRNA mediated down regulation. Data shows the average expression in 5 independent samples and error bars indicate SEM. * $P < 0.05$ (paired T-test). (**c**) Diagonal plot indicating significant down- (*NOTCH1*, *STAT5A*, *PTCRA*) (*blue*) or upregulation (*IKZF1*, *RAG1*) (*red*) of genes upon PHF6 knockdown in ALL-SIL lymphoblasts. (**d**) Gene Set Enrichment Analysis shows that the top-500 significantly induced genes in CB $CD34^+$ progenitors cultured on an OP9-DLL1 stromal feeder layer in comparison to OP9-GFP cocultures are significantly enriched in the set of genes that are downregulated upon knockdown of PHF6 in ALL-SIL T-ALL cells.

Supplementary Figure 3: The development of CD4+CD8β+ DP thymocytes after (**a**) 6 days of coculture or (**b**) 14 days of coculture of control versus *PHF6* shRNA transduced or DMSO versus 1 µM GSI treated CD34+ thymocytes in OP9-DLL1 cocultures in the presence of IL7, SCF and FLT3L. Left panels show flow cytometry analysis while right panels show the absolute cell counts (*$p<0.05$ Wilcoxon signed rank test).

Supplementary Figure 4: (**a**) PHF6 protein sequence alignment between human, zebrafish, mouse, chicken, chimpanzee and rat shows high conservation throughout evolution. Percentages in blue indicate percentage peptide identity compared to human PHF6. PHD = plant homeodomain, NLS = nuclear localization signal. (**b**) Normalized *phf6* expression in various zebrafish organs as indicated. Error bars indicate SEM of 2 biological replicates. (**c**) Sequence of the 2nd exon of Phf6 with TALEN induced mutations indicated on DNA and protein level. (**d**) RT-qPCR phf6 down regulation. $phf6^{c.165del10/+}$ zebrafish show a 60% reduction of phf6 expression compared to wild type (ab). (**e**) Whole-mount *in situ* hybridization assay shows statistical significant higher *rag1* expression in phf6 morphant fish (splice site morpholino) (n=83) versus control (n=87) zebrafish of 4 dpf (p=0,0117, Fisher exact).

Supplementary Tables

Supplementary Table 1: List of all leading edge genes significantly enriched upon scoring of a NOTCH1 gene signature, induced in CB CD34+ progenitors cultured on an OP9-DLL1 versus OP9-GFP stromal feeder layer, in Jurkat cells with stable PHF6 knockdown using Gene Set Enrichment analysis.

GENE SYMBOL	GENE_TITLE	RANK IN GENE LIST
CCR8	chemokine (C-C motif) receptor 8	1
GIMAP4	GTPase, IMAP family member 4	13
ANXA3	annexin A3	23
FAM46C	family with sequence similarity 46, member C	26
MYO7B	myosin VIIB	35
OAS3	2'-5'-oligoadenylate synthetase 3, 100kDa	62
IGLL1	immunoglobulin lambda-like polypeptide 1	80
GBP1	guanylate binding protein 1, interferon-inducible, 67kDa	85
ADAMTS1	ADAM metallopeptidase with thrombospondin type 1 motif, 1	91
PRDM1	PR domain containing 1, with ZNF domain	111
GIMAP1	GTPase, IMAP family member 1	115
GIMAP7	GTPase, IMAP family member 7	179
HSH2D	hematopoietic SH2 domain containing	180
MYO1A	myosin IA	192
TMEM65	transmembrane protein 65	233
CTSG	cathepsin G	247
LAPTM4B	lysosomal associated protein transmembrane 4 beta	256
RASAL1	RAS protein activator like 1 (GAP1 like)	262
RGPD1	RANBP2-like and GRIP domain containing 1	268
NPPC	natriuretic peptide precursor C	280
GRB10	growth factor receptor-bound protein 10	293
STC2	stanniocalcin 2	295
CR2	complement component (3d/Epstein Barr virus) receptor 2	300
TMEM92	transmembrane protein 92	302
VDR	vitamin D (1,25- dihydroxyvitamin D3) receptor	306
PMAIP1	phorbol-12-myristate-13-acetate-induced protein 1	310
GAD1	glutamate decarboxylase 1 (brain, 67kDa)	313
PCBP3	poly(rC) binding protein 3	326
P2RX5	purinergic receptor P2X, ligand-gated ion channel, 5	343
EGR1	early growth response 1	345
ASCL2	achaete-scute complex-like 2 (Drosophila)	395
SH3PXD2B	SH3 and PX domains 2B	397
SDSL	serine dehydratase-like	402
GIMAP5	GTPase, IMAP family member 5	418
OSGIN1	oxidative stress induced growth inhibitor 1	420
SLC16A9	solute carrier family 16, member 9 (monocarboxylic acid transporter 9)	435
GIMAP6	GTPase, IMAP family member 6	455

CHRNA6	cholinergic receptor, nicotinic, alpha 6	507
ANXA2	annexin A2	515
FAM83F	family with sequence similarity 83, member F	538
GIMAP2	GTPase, IMAP family member 2	554
CYP2S1	cytochrome P450, family 2, subfamily S, polypeptide 1	564
BCL2A1	BCL2-related protein A1	569
TREML2	triggering receptor expressed on myeloid cells-like 2	580
LIMS2	LIM and senescent cell antigen-like domains 2	613
DTX1	deltex homolog 1 (Drosophila)	622
COL27A1	collagen, type XXVII, alpha 1	624
SLC7A11	solute carrier family 7, (cationic amino acid transporter, y+ system) member 11	633
IFIT1	interferon-induced protein with tetratricopeptide repeats 1	658
ROPN1L	ropporin 1-like	694
RGS9	regulator of G-protein signalling 9	723
TUBB6	tubulin, beta 6	729
OSBPL6	oxysterol binding protein-like 6	732
TNFSF10	tumor necrosis factor (ligand) superfamily, member 10	738
RAI14	retinoic acid induced 14	747
OSM	oncostatin M	809
TBX21	T-box 21	811
ANGPTL6	angiopoietin-like 6	812
PRR5	proline rich 5 (renal)	815
CTSH	cathepsin H	918
KIAA0125	KIAA0125	919
FAM27A	family with sequence similarity 27, member A	943
STON2	stonin 2	958
GNGT2	guanine nucleotide binding protein (G protein), gamma transducing activity polypeptide 2	993
SLC35F3	solute carrier family 35, member F3	1001
POLR3G	polymerase (RNA) III (DNA directed) polypeptide G (32kD)	1005
TSPAN9	tetraspanin 9	1055
TNFRSF12A	tumor necrosis factor receptor superfamily, member 12A	1057
GPR68	G protein-coupled receptor 68	1071
IL15	interleukin 15	1109
CD1B	CD1b molecule	1126
TFRC	transferrin receptor (p90, CD71)	1170
OASL	2'-5'-oligoadenylate synthetase-like	1211
DENND3	DENN/MADD domain containing 3	1233
APOL1	apolipoprotein L, 1	1262
EIF4EBP1	eukaryotic translation initiation factor 4E binding protein 1	1295
ATF3	activating transcription factor 3	1335
IFITM1	interferon induced transmembrane protein 1 (9-27)	1385
CCL1	chemokine (C-C motif) ligand 1	1389
TSPAN15	tetraspanin 15	1410
ARHGEF17	Rho guanine nucleotide exchange factor (GEF) 17	1419
HSPA4L	heat shock 70kDa protein 4-like	1425

PLAU	plasminogen activator, urokinase	1453
MYO5C	myosin VC	1459
PCGF5	polycomb group ring finger 5	1506
NRP1	neuropilin 1	1637
PHOSPHO1	phosphatase, orphan 1	1670
PSAT1	phosphoserine aminotransferase 1	1743
EPB49	erythrocyte membrane protein band 4.9 (dematin)	1752
SHQ1	SHQ1 homolog (S. cerevisiae)	1810
ICA1	islet cell autoantigen 1, 69kDa	1811
ELL2	elongation factor, RNA polymerase II, 2	1818
WT1	Wilms tumor 1	1902
NETO2	neuropilin (NRP) and tolloid (TLL)-like 2	1936
SNX25	sorting nexin 25	1953
IFITM3	interferon induced transmembrane protein 3 (1-8U)	1965
GNAO1	guanine nucleotide binding protein (G protein), alpha activating activity polypeptide O	2003
ALDH1B1	aldehyde dehydrogenase 1 family, member B1	2012
TLR5	toll-like receptor 5	2013
UPP1	uridine phosphorylase 1	2051
HES4	hairy and enhancer of split 4 (Drosophila)	2053

Supplementary Table 2: T cell development with GSI treatment in wild type (ab) zebrafish

	**4 dpf**			**5 dpf**			**6 dpf**		
	DMSO	2 μM	8 μM	DMSO	2 μM	8 μM	DMSO	2 μM	8 μM
# GFP +	6/40	26/44	26/36	35/40	41/44	36/36	39/39	43/43	16/16
%GFP +	15%	59%	72%	88%	93%	100%	100%	100%	100%
Mean thymus size (μM)	58	354	515	738	988	807	1244	1416	604

Supplementary Table 3: statistical analysis comparing thymus size GSI treatment on wild type (ab) zebrafish

		P-value (Wilcoxon rank sum test)	P adjusted (FDR)
4 dpf	DMSO vs 2 μM	**2.865e-05**	**5.7300e-05**
	DMSO vs 8 μM	**1.844e-07**	**1.1064e-06**
5 dpf	DMSO vs 2 μM	**0.0452**	6.6924e-02
	DMSO vs 8 μM	0.5632	5.6320e-01
6 dpf	DMSO vs 2 μM	0.05577	6.6924e-02
	DMSO vs 8 μM	**6.497e-07**	**1.9491e-06**

Supplementary Table 4: T cell development in wild type (ab) versus phf6 mutant (*phf6*$^{c.165del10/+}$) zebrafish

	**4 dpf**		**5 dpf**		**6 dpf**	
	AB	*phf6*$^{c.165del10/+}$	*AB*	*phf6*$^{c.165del10/+}$	*AB*	*phf6*$^{c.165del10/+}$
# GFP^{+} zebrafish	9/65	34/55	46/65	53/55	65/65	55/55
% GFP^{+} zebrafish	13,85 %	61,82 %	70,77 %	96,36 %	100 %	100 %
Mean thymus Size (µm)	75,74	389,48	736,47	1435,50	1559,77	1492,31
P value (Wilcoxon rank sum test) comparing thymus size	**1.655e-07**		**9.139e-09**		0.5205	

Supplementary Methods

RNA-isolation, cDNA synthesis and RT-qPCR

Total RNA was isolated using the miRNeasy mini kit (Qiagen) with DNA digestion on-column. By means of spectrophotometry, RNA concentrations were measured (Nanodrop 1000) and RNA integrity was evaluated (Experion, Bio-Rad). Next, cDNA synthesis was performed using the iScript cDNA synthesis Kit (Bio-Rad) followed by RT-qPCR using the LightCycler 480 (Roche) and the following primers: *PHF6*-Fw: AAAAGGGCCTACAAGACAG; *PHF6*-Rev: ACAATGGCACAAAGAACAC; *NOTCH1*-Fw: GCAGTTGTGCTCCTGAAGAA; *NOTCH1*-Rev: CGGGCGGCCAGAAAC; *DTX1*-Fw: ACGAGAAAGGCCGGAAGGT; *DTX1*-Rev: GGTGTTGGACGTGCCGATAG; the normalization genes *TBP* and *YWHAZ* were selected using GeNorm (Vandesompele et al. 2002).

Western blotting

SDS-PAGE was performed according to standard protocols. For immunoblotting, following antibodies were used: rabbit polyclonal antibody to PHF6 (1:2000, Bethyl Laboratories, A301-451A) and mouse monoclonal antibody to alpha-tubulin (1:2000, Sigma-Aldrich, T5168). Protein level quantification was performed using the ImageJ software.

siRNA mediated knockdown of *PHF6* in ALL-SIL T-ALL lymphoblasts

ALL-SIL T-ALL cells were electroporated (250 V, 1000 µF) using a Genepulser Xcell device (Biorad) with 400 nM of Silencer Select Negative Control 1 siRNA (Ambion, #AM4635) or siRNAs targeting *PHF6* (Silencer Select, Ambion, #4392420, s38848 and ON-TARGETplus SMARTpool; Dharmacon, Lafayette, CO, USA). ALL-SIL cells were collected 96h post-electroporation.

Gene expression profiling and Gene Set Enrichment Analysis

RNA samples were profiled on a custom designed Agilent micro-array covering all protein coding genes (33,128 mRNA probes, Human Sureprint G3 8x60k micro-arrays (Agilent)) and 12,000 lncRNAs (23,042 unique lncRNA probes) (Volders et al. 2013). In total, 3 independent samples from each control and *PHF6* shRNA transduced $CD34^+$ CB HPCs from OP9-GFP cocultures and 3 independent samples from each control and *PHF6* shRNA transduced $CD34^+$ CB HPCs from OP9-DLL1 cocultures were profiled 72 hours following transduction. In addition, 3 independent samples from control and *PHF6* siRNA transfected (electroporation) ALL-SIL cells and 3 independent samples from control and *PHF6* shRNA transduced Jurkat cells were profiled 96 hours following electroporation/transduction using the same platform. Expression data were normalized using the VSN-package (Bioconductor release 2.12) in R. The expression datasets generated are deposited in the Gene Expression Omnibus database (GEO) (GSE85373). Differential expression analysis was performed in R using Limma. Public datasets (GSE24759) were normalized using the Affy-package (Bioconductor release 2.12) in R. Gene Set Enrichment Analysis (GSEA) (Subramanian et al. 2005) was used to score our gene sets compiled from the publically available gene expression data (Novershtern et al. 2011).

Zebrafish maintenance

Zebrafish were housed in a Zebtec semi-closed recirculation housing system (Techniplast, Italy) and kept at a constant pH (7.5), temperature (27–28 °C) and conductivity (500 µS) on a 14/10 light/dark cycle. Wild type AB and *Tg(rag2:GFP)* zebrafish were obtained from the Zebrafish International Resource Center (ZIRC). Phf6 knock-out zebrafish were generated by injection of 125 pg of TALEN RNA for each arm as described by Moore et al., introducing a 10 bp deletion in exon 2 (c.165del10) (Moore et al. 2012). Founders were screened by microsatellite PCR and then confirmed by Sanger sequencing. The zebrafish line was further

outcrossed twice to AB or Casper line before functional analysis. Approval for this study was provided by Massachusetts General Hospital Subcommittee on Research Animal Care (OLAW Assurance # A3596-01 under protocol #2011N000127) and by the Ghent University committee on Ethics of Animal Experiments (Ghent University Hospital, Ghent, Belgium; Permit Number: ECD 11/37). All efforts were made to minimize pain and discomfort.

Gene expression analysis on isolated zebrafish thymocytes

Heterogenous *Tg(rag2:GFP)* and *Tg(rag2:GFP);phf6*$^{c.165del10/+}$ zebrafish embryos were raised until 6 dpf. 70-100 embryos were killed by an overdose of tricaine and dissociated by adding a pre-heated trypsin (0,25% trypsin, 1 mM EDTA) solution and incubating them for 90 min at 28.5°C. During incubation, the embryos were grinded by pipetting up and down every 15 min. The reaction was stopped by adding $CaCl_2$ and FCS to a final concentration of 10 mM and 10% respectively. The obtained cells were pelleted (5 min x 800g), washed with PBS and resuspended in a resuspension buffer (Leibovitz's L-15 medium + L-Glutamine without Phenol Red, FCS 10 %, 0.8 mM CaCl 2 penicillin 50 U/µL, streptomycin 0.05 mg/mL). The cells were filtered several times through a 40 µM mesh strainer and flow cytometry mediated cell sorting was immediately proceeded (BD FACS ARIA III, Biosciences). 30 000 GFP^+ thymocytes were sorted directly into the lysis buffer of the Qiagen RNeasy plus micro kit (350 µl buffer RTL supplemented with 3.5 µl 2-mercapto-ethanol). RNA isolation was performed according to the manufacturer's guidelines. Quality of the obtained RNA was analyzed on the fragment analyzer (High Sensitivity RNA Analysis Kit, DNF-472-0500). Dnase treatment was performed by the use of the Heat&Run gDNA removal kit (ArticZymes). cDNA was synthetized and amplified with the SMART-seq V4 ultra low Input RNA kit (Clontech) and used for subsequent RT-qPCR (Loontiens et al. 2019). Following primers were used for expression analysis: fw *phf6:* GCAGTGATGATGAACAGGGA; rev *phf6:* CTGTATCGTCATTGCCTTGC; fw *Notch1a:* CGAACTGCCAGATGAACATT; rev *Notch1a:* TTTACAGGGACGTGGAGAA. The expressed repeat elements (ERE's) hadn10, loopern4, tdr7 were used for normalization (Vanhauwaert et al. 2014).

Adult zebrafish were processed similarly. Thymi of 6 adult *Tg(rag2:GFP)* and *Tg(rag2:GFP); phf6*$^{c.165del10/+}$ zebrafish were dissected based on GFP signal. This tissue was collected in 0.9xPBS, 5%FBS and mechanically disrupted by pipetting and filtering through a 40µM mesh strainer. GFP^+ thymocytes were sorted, RNA isolated with the RNeasy plus micro kit (Qiagen) and cDNA synthetized with the iScript advanced kit (BioRad) and subsequently used for RT-qPCR.

Zebrafish organ dissection

Adult zebrafish (>3 months) were euthanized with 0.4% tricaine methanesulfonate and different organs were dissected (brain, eye, intestine, kidney, liver, testis, thymus and ovaria). RNA was isolated from the different organs using the QIAGEN miRNAeasy micro kit with an on-column DNAseI treatment using the RNase-Free DNase set (Qiagen) according to the manufacturer's guidelines. A whole transcriptome amplification of these samples was executed as previously described (NuGEN) (Vermeulen et al. 2009). cDNA was synthesized via the Bio-Rad iScript Advanced kit. For the RT-qPCR reaction, 2.5 ml SsoAdvanced SYBRN Green supermix (Bio-Rad) was mixed with 5 ng cDNA and 250 nM of forward and reverse primer in a 384 well plate (Bio-Rad) and run on a lightcycler 480. The expressed repeat elements (ERE's) hadn10, loopern4, tdr7 were used for normalization (Vanhauwaert et al. 2014). RT-qPCR data were analyzed with qBase+. The average expression of 3 primer sets (fw1: GAGCTTCAGCACGTCTTCGG; rev1: AGGCTGATGAGAATAGCATGCAC; fw2: GCAGTGATGATGAACAGGGA; rev2: CTGTATCGTCATTGCCTTGC; fw3: GAGGAATTTACAAGCTGTATTGT; rev3: TCTTCATGCACCATCTCCTA) targeting *phf6*

was used for expression analysis. For Notch1a expression analysis following primers were used: fw *Notch1a*: CGAACTGCCAGATGAACATT; rev *Notch1a*: TTTACAGGGACGTGGAGAA.

Zebrafish morpholino injection and *in situ* hybridization

6.5 ng of *phf6* splice site morpholino (5'-TGTACAGCTAACATACCATGCACTT-3') or non-targeting control was injected in 1-cell stage embryos. At 4 days post fertilization, the embryos were collected for whole mount *in situ* hybridization (WISH) as described by Thisse et al.(Thisse and Thisse 2008) to visualize *rag1* expression. RNA antisense probes were generated by ligating the T3 promotor to the reverse primer. Following primers were used: Fw: F-CACCATGTCGACACCTGTTC, R-GGATCCATTAACCCTCACTAAAGGGAATGACAGTGAAGCGCATAAGG. *In vitro* transcription was performed by the use of T3 RNA polymerase (Promega) and DIG RNA labeling mix (Roche) according to manufacturer's instructions.

Supplementary References

Canté-Barrett, Kirsten, Rui D. Mendes, Yunlei Li, Eric Vroegindeweij, Karin Pike-Overzet, Tamara Wabeke, Anton W. Langerak, Rob Pieters, Frank J.T. Staal, and Jules P.P. Meijerink. 2017. "Loss of CD44dim Expression from Early Progenitor Cells Marks T-Cell Lineage Commitment in the Human Thymus." *Frontiers in Immunology* 8 (JAN). https://doi.org/10.3389/fimmu.2017.00032.

Casero, David, Salemiz Sandoval, Christopher S. Seet, Jessica Scholes, Yuhua Zhu, Vi Luan Ha, Annie Luong, Chintan Parekh, and Gay M. Crooks. 2015. "Long Non-Coding RNA Profiling of Human Lymphoid Progenitor Cells Reveals Transcriptional Divergence of B Cell and T Cell Lineages." *Nature Immunology* 16 (12): 1282–91. https://doi.org/10.1038/ni.3299.

Loontiens, Siebe, Lisa Depestel, Suzanne Vanhauwaert, Givani Dewyn, Charlotte Gistelinck, Karen Verboom, Wouter Van Loocke, et al. 2019. "Purification of High-Quality RNA from a Small Number of Fluorescence Activated Cell Sorted Zebrafish Cells for RNA Sequencing Purposes." *BMC Genomics* 20 (1): 228. https://doi.org/10.1186/s12864-019-5608-2.

Moore, Finola E., Deepak Reyon, Jeffry D. Sander, Sarah A. Martinez, Jessica S. Blackburn, Cyd Khayter, Cherie L. Ramirez, J. Keith Joung, and David M. Langenau. 2012. "Improved Somatic Mutagenesis in Zebrafish Using Transcription Activator-Like Effector Nucleases (TALENs)." Edited by Joshua L. Bonkowsky. *PLoS ONE* 7 (5): e37877. https://doi.org/10.1371/journal.pone.0037877.

Novershtern, Noa, Aravind Subramanian, Lee N Lawton, Raymond H Mak, W Nicholas Haining, Marie E McConkey, Naomi Habib, et al. 2011. "Densely Interconnected Transcriptional Circuits Control Cell States in Human Hematopoiesis." *Cell* 144 (2): 296–309. https://doi.org/10.1016/j.cell.2011.01.004.

Subramanian, Aravind, Pablo Tamayo, Vamsi K. Mootha, Sayan Mukherjee, Benjamin L. Ebert, Michael A. Gillette, Amanda Paulovich, et al. 2005. "Gene Set Enrichment Analysis: A Knowledge-Based Approach for Interpreting Genome-Wide Expression Profiles." *Proceedings of the National Academy of Sciences of the United States of America* 102 (43): 15545–50. https://doi.org/10.1073/pnas.0506580102.

Thisse, Christine, and Bernard Thisse. 2008. "High-Resolution in Situ Hybridization to Whole-Mount Zebrafish Embryos." *Nature Protocols* 3 (1): 59–69. https://doi.org/10.1038/nprot.2007.514.

Vandesompele, Jo, Katleen De Preter, Filip Pattyn, Bruce Poppe, Nadine Van Roy, Anne De Paepe, and Frank Speleman. 2002. "Accurate Normalization of Real-Time Quantitative RT-PCR Data by Geometric Averaging of Multiple Internal Control Genes." *Genome Biology* 3 (7): research0034.1. https://doi.org/10.1186/gb-2002-3-7-research0034.

Vanhauwaert, Suzanne, Gert Van Peer, Ali Rihani, Els Janssens, Pieter Rondou, Steve Lefever, Anne De Paepe, et al. 2014. "Expressed Repeat Elements Improve RT-QPCR Normalization across a Wide Range of Zebrafish Gene Expression Studies." Edited by Nicholas S. Foulkes. *PLoS ONE* 9 (10): e109091. https://doi.org/10.1371/journal.pone.0109091.

Vermeulen, Joëlle, Stefaan Derveaux, Steve Lefever, Els De Smet, Katleen De Preter, Nurten Yigit, Anne De Paepe, Filip Pattyn, Frank Speleman, and Jo Vandesompele. 2009. "RNA Pre-Amplification Enables Large-Scale RT-QPCR Gene-Expression Studies on Limiting Sample Amounts." *BMC Research Notes* 2 (November): 235. https://doi.org/10.1186/1756-0500-2-235.

Volders, Pieter Jan, Kenny Helsens, Xiaowei Wang, Björn Menten, Lennart Martens, Kris

Gevaert, Jo Vandesompele, and Pieter Mestdagh. 2013. "LNCipedia: A Database for Annotated Human lncRNA Transcript Sequences and Structures." *Nucleic Acids Research* 41 (D1). https://doi.org/10.1093/nar/gks915.
