# Supplementary figures and images for: PHF6 Expression Levels Impact Human Hematopoietic Stem Cell Differentiation
Source: Front Cell Dev Biol. 2020 Nov 4;8:599472. doi: 10.3389/fcell.2020.599472 (PMC7672048; doi:10.3389/fcell.2020.599472)

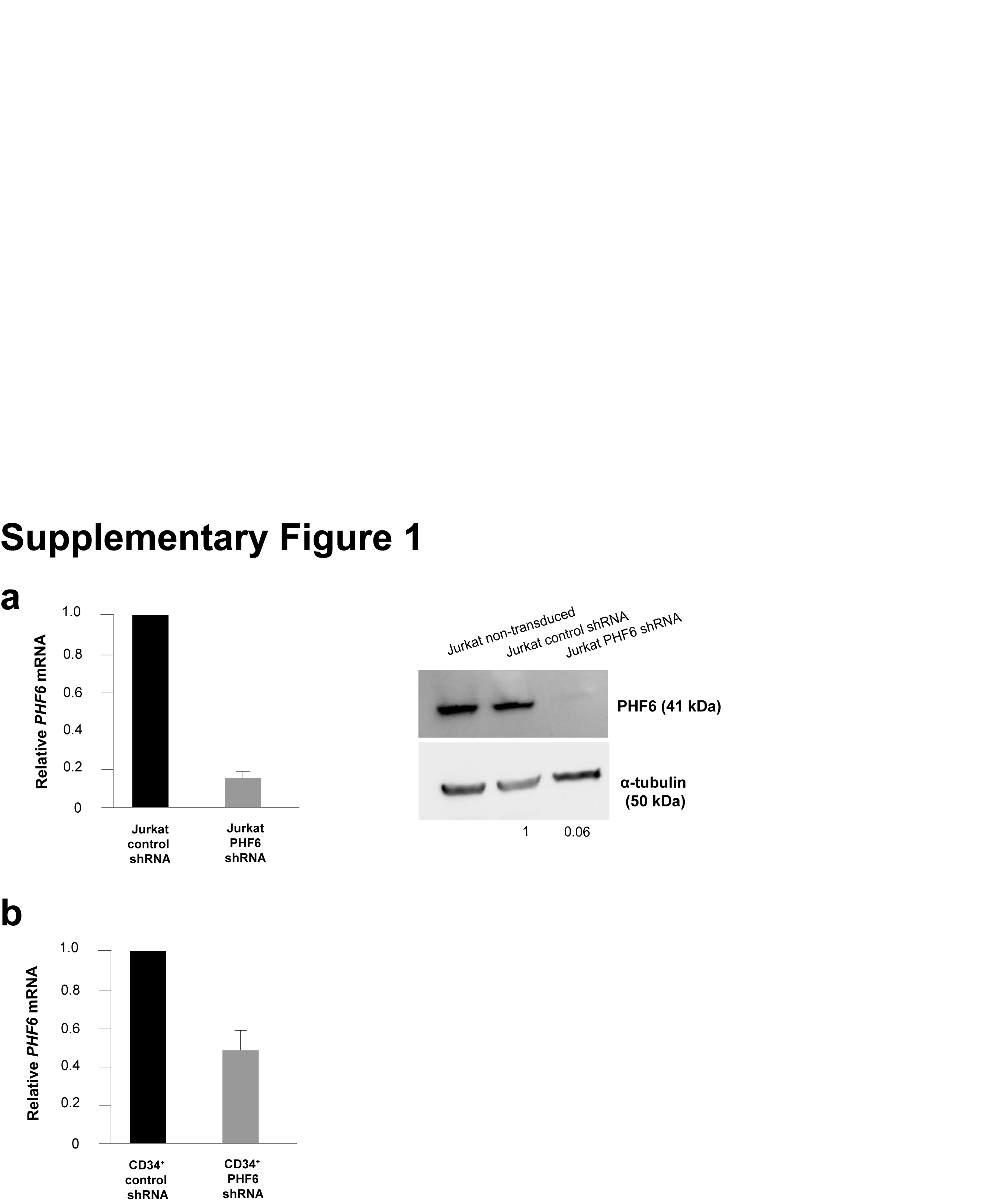

Supplement: Supplementary file 2 [file Image_1.JPEG]

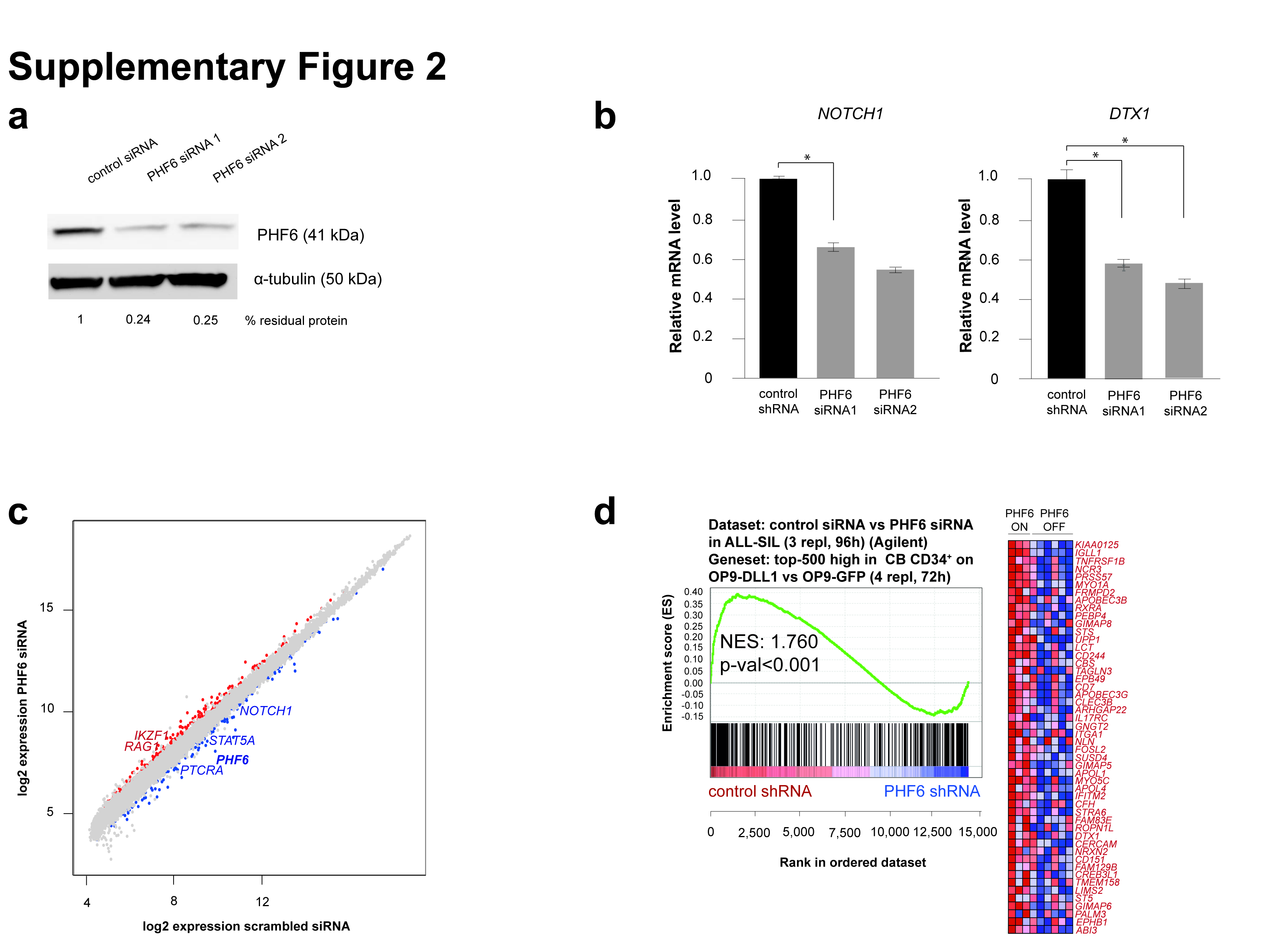

Supplement: Supplementary file 3 [file Image_2.JPEG]

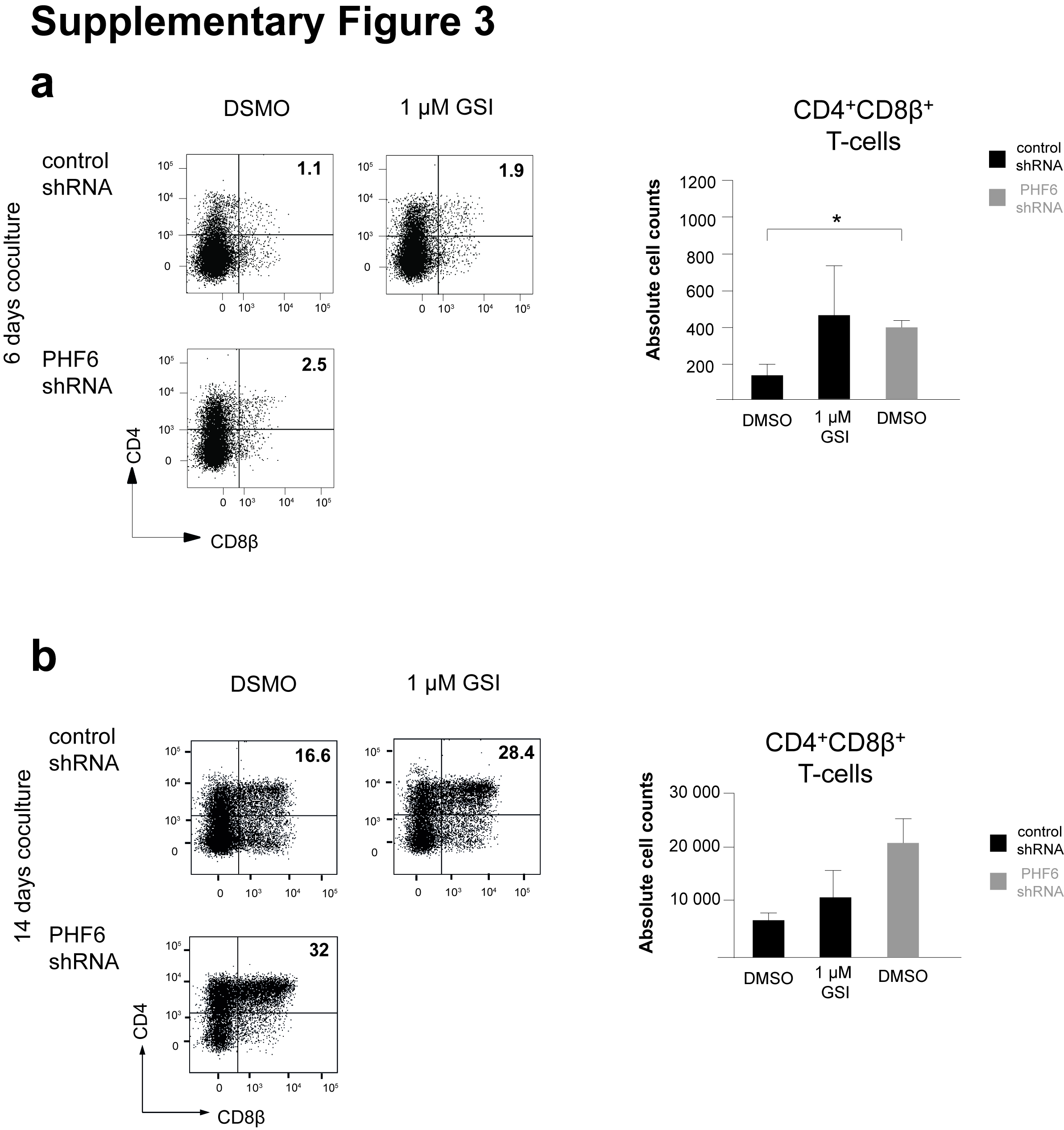

Supplement: Supplementary file 4 [file Image_3.JPEG]

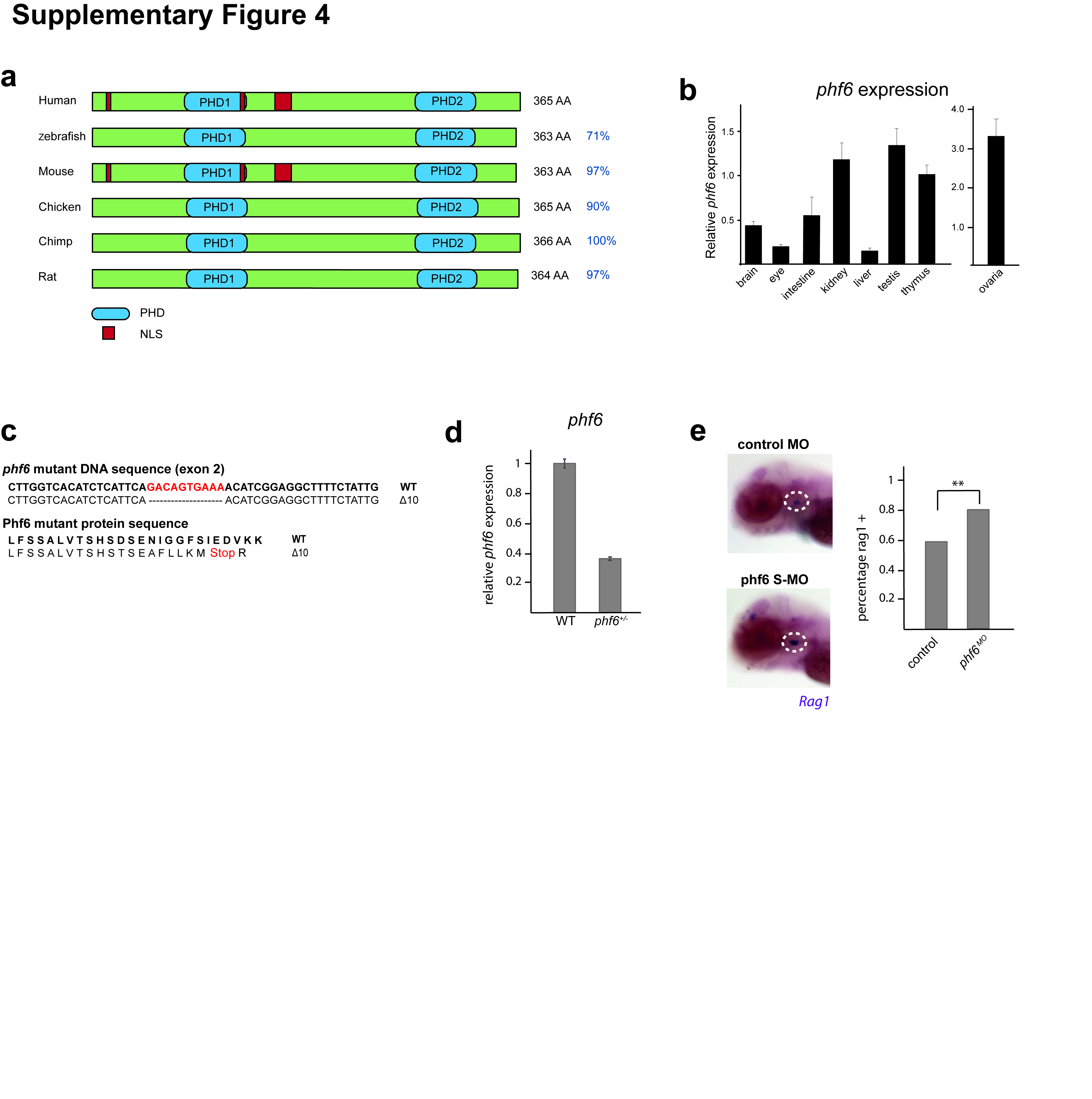

Supplement: Supplementary file 5 [file Image_4.JPEG]
